# The longitudinal predictive effect of self-reported frequency of premenstrual syndrome on depression: Findings from the Australian Longitudinal Study on Women's Health

**DOI:** 10.3389/fpubh.2023.1126190

**Published:** 2023-03-23

**Authors:** Lulu Hou, Lele Chen, Wenpei Zhang

**Affiliations:** ^1^Department of Psychology, Shanghai Normal University, Shanghai, China; ^2^School of Education Science, Nantong University, Nantong, China; ^3^Department of Business Administration, School of Business, Anhui University of Technology, Maanshan, China

**Keywords:** premenstrual syndrome, PMS, depression, risk factor, logistic regression

## Abstract

**Background:**

Previous studies have revealed a high comorbidity between premenstrual syndrome (PMS) and depression; however, whether PMS can longitudinally predict depression has not been examined in large sample studies.

**Methods:**

This study surveyed 8,133 women from the 1973–78 cohort of the Australian Longitudinal Study on Women's Health. Participants completed seven repeated measurements at 3-year intervals starting in 2000 (aged 22–27 years). Binary logistic and multivariate ordered logistic regression models were used to examine the predictive role of self-reported frequency of PMS symptoms in 2000 on self-reported diagnosis of depression and frequency of depressive symptoms, respectively, for each follow-up survey.

**Results:**

Self-reported frequency of PMS symptoms in the year 2000 predicted self-reported diagnosis of depression in most follow-up surveys. Specifically, compared to women who reported “never” had PMS symptoms in 2000, those who reported “often” had them were more likely to report a diagnosis of depression in 2006 (OR = 1.72), 2012 (OR = 1.88), 2015 (OR = 1.49), and 2018 (OR = 1.90); and those who reported “sometimes” had PMS symptoms in 2000 were more likely to report a diagnosis of depression in 2012 (OR =1.37) and 2018 (OR = 1.59). Furthermore, self-reported frequency of PMS symptoms in 2000 predicted self-reported frequency of depressive symptoms in each follow-up survey. Compared to women who reported “never” had PMS symptoms in 2000, those who reported “sometimes”, or “often”, had PMS symptoms reported depressive symptoms more frequently.

**Conclusion:**

Self-reported frequency of PMS can predict the self-reported frequency of depressive symptoms and the subsequent diagnosis of depression.

## 1. Introduction

According to The Global Burden of Diseases, Injuries, and Risk Factors Study (GBD) 2019, depression was the second highest ranked disability among all mental disorders (1), with annual increases since 1990. The disability-adjusted life year for depression puts it in the top 10 disorders among individuals aged 10–49 years old (2). The results of the meta-analysis by Lim et al. (3) showed that between 1994 and 2014, the lifetime prevalence of depression was ~10.8%, and the prevalence was significantly higher in women than in men. Other studies have further that the sex difference in prevalence of depression begins mainly in mid-adolescence and decreases after 55 years of age (4–6). There was a stable prevalence of depression in males from the age of 13 years, while the prevalence of depression in females increases yearly and is about twice as high as that of males (7, 8). Researchers have proposed several models to explain this sex difference in terms of physiological, psychological, and social factors (9, 10). Because this sex differences begin at puberty and disappear at menopause, and changes in estrogen and progesterone cause changes in neural networks related to emotions (11), some researchers have suggested that sex differences in depression are related to the cyclical fluctuations of hormones in women (7, 10, 12, 13).

Premenstrual syndrome (PMS) is an emotional condition associated with hormonal fluctuations during the menstrual cycle and refers to a series of physical, psychological, and behavioral symptoms that occur before the onset of menstruation and improve, or disappear, after the onset (14). Previous studies have shown that, although PMS is independent of depression, rather than a variant of it (15), they share some common risk factors (e.g., history of abuse, elevated perceived stress, and unhealthy lifestyle) (16–24) and symptoms (e.g., depressed mood, feelings of hopelessness, and decreased interest in activities) (25); this has led some researchers to examine the comorbidity between them. For example, Yonkers et al. (26) found that 22% of women with PMS had comorbid major depressive disorder (MDD) and another 5.4% had mild depressive disorder. Forrester-Knauss et al. (27) conducted a survey of 3,518 women in Switzerland and found that 11.3% of women with moderate PMS and 24.6% of women with severe PMS had MDD. Sadr et al. (28) showed that 70% of women with PMS had varying degrees of depressive symptoms in a survey of 100 medical interns, while only 40% of women who did not have PMS suffered from varying degrees of depressive symptoms. In addition, Padhy et al. (23) also found that the diagnosis of depression was associated with the severity of PMS symptoms. Further, because PMS and its severe form, premenstrual dysthymic disorder (PMDD), usually precede depression, researchers have viewed PMS as another risk factor, independent of a family history of depression, arguing that PMDD is the initial stage of disorders triggered by the menstrual cycle, while depression may culminate after the accumulation of disorders predicted by PMDD (29, 30).

However, all of the above studies have limitations: first, most of them used convenience sampling, selecting as participants patients coming to the hospital (26), medical students (28), or nursing school students and staff (23), and only one study used a large representative sample of women (27); second, previous studies examined the comorbidity of PMS and depression primarily using cross-sectional designs (23, 26–28). One early study used a longitudinal approach and found that the proportion of women with PMDD who developed depression at 2 years was 14 times higher than that of women without PMDD; however, its sample only included eight women with, and nine women without, PMDD (31). Combining these limitations of sample selection and study design, it is unclear whether PMS can be used as a risk factor for depression; in other words, whether PMS can longitudinally predict depression, and if so, how long this effect lasts. Furthermore, while previous studies have used whether diagnostic criteria are met as the dependent variable, a growing body of research supports viewing mental disorders as a continuum (32, 33), and argues for attention and early intervention for people with some depressive symptoms who do not meet diagnostic criteria for depression (34).

To address these gaps, the present study used the prospective Australian Longitudinal Study on Women's Health (ALSWH), a cohort of women aged 22–27 years, with 18 years of follow-up, to examine the longitudinal predictive relationship of self-reported frequency of PMS symptoms to depression and to include both self-reported diagnosis of depression and frequency of depressive symptoms in the investigation. Based on the results of previous studies (23, 26–28), we hypothesized that the self-reported frequency of PMS symptoms at the baseline would predict the self-reported frequency of depressive symptoms and the self-reported diagnosis of depression in the follow-up surveys.

## 2. Materials and methods

### 2.1. Participants

This study used data from the 1973–78 cohort of the Australian Longitudinal Study on Women's Health (ALSWH). The ALSWH included more than 40,000 women from three cohorts born in 1921–26, 1946–51, and 1973–78; initial intake took place in 1996 with follow-up surveys conducted at ~3-year intervals (35). More recently, in 2012, they included more than 10,000 women born in 1989–95 and followed up at yearly intervals. The database covers all women in Australia (including those living in Australia, but without permanent residency) and is broadly representative of the Australian population. Each survey examined participants' physical and mental health status, exercise and diet, health care, and substance use *via* a series of questionnaires and questions in addition to collecting demographic variables (www.alswh.org.au/forresearchers/surveys) (36).

This study used data from the 1973–78 cohort, and included participants from eight surveys conducted in 1996 (*n* = 14,247), 2000 (*n* = 9,688), 2003 (*n* = 9,081), 2006 (*n* = 9,145), 2009 (*n* = 8,199), 2012 (*n* = 8,009), 2015 (*n* = 7,186), and 2018 (*n* = 7,121). Because Survey 1 did not collect depression-related data, we used the data from Survey 2 (conducted in 2000 when women aged 22–27 years) to Survey 8 (conducted in 2018 when women aged 40–45 years) for analysis, and used the data from Survey 2 as the baseline.

### 2.2. Measurements

#### 2.2.1. Self-reported premenstrual syndrome

The presence of PMS was reported in each survey using a 4-point Likert scale ranging from 0 to 3: “In the past 12 months, have you had premenstrual tension? (never, rarely, sometimes, or often).”

#### 2.2.2. Self-reported diagnosis of depression

Depression diagnostic status was reported in each survey using a dichotomous question: “In the last 3 years, have you ever been diagnosed with, or treated for, depression (non-postpartum)? (yes or no).” Since the interval between Survey 1 and Survey 2 is 4 years, this question asked about the last 4 years in 2000 (i.e., Survey 2).

#### 2.2.3. Self-reported depressive symptoms

The presence of depressive symptoms was reported in each survey using a 4-point Likert scale ranging from 0 to 3: “In the past 12 months, have you had depression? (never, rarely, sometimes, often).”

### 2.3. Covariates

The following four categories of variables associated with PMS and depression were included as covariates: (1) demographic variables: including age, highest educational qualification (no formal qualification, year 10, year 12, trade/apprenticeship, certificate/diploma, university degree, or higher university degree); marital status (married, de facto marriage, not married, separated, or widowed); residence (major cities, inner regional, outer regional, or remote/very remote); and BMI (<18.5, 18.5 to < 25, 25 to < 30, or ≥30); (2) lifestyle habits: sleep difficulties (never, rarely, sometimes, or often); physical activity (sedentary, low, moderate, or high); smoking (never smoked, ex-smoker, smoke < 10 day, smoke 10–19 day, or smoke ≥20 day), alcohol consumption (low risk drinker, non- drinker, non-drinker, rarely drinker, risky drinker, or high risk drinker), oral contraceptive pills (OCPs) use (two dichotomous questions examining use of oral pills for contraception, and for other reasons); (3) menstrual symptoms: irregular menstrual periods (never, rarely, sometimes, or often); severe period pain (never, rarely, sometimes, or often); and heavy periods (never, rarely, sometimes, or often); and (4) other variables: history of abuse (four dichotomous questions were used to examine physical abuse, emotional abuse, sexual abuse, and harassment) and perceived stress (using the Perceived Stress Scale (PSS) developed by Bell and Lee (37), which contains twelve 5-point Likert items ranging from 0, “not at all stressed,” to 4, “extremely stressed,” to examine participants' stress from family members' health, school, work, etc.).

It is important to note that for some variables, there were small numbers of participants in some categories; in such cases, we merged the categories, following examples from previous studies (38–40). “Highest educational qualification” was combined into: up to high school or equivalent, trade/diploma, and university degree or higher; “marital status” was combined into: married/de facto and not married/separated; “residence” was combined into: major cities, inner regional, and outer regional/remote/very remote; “smoking” was combined into: never smoker, ex-smoker, and current smoker; and “alcohol consumption” was combined into: never drinker/rarely drinker, low risk drinker, and risky/high risk drinker; the two questions on OCPs were combined into: user and non-user. Furthermore, the mean score of the PSS was divided into the categories: not at all stressed (0), somewhat stressed (0–1), moderately stressed (1–2), very stressed (2–3), and extremely stressed (3–4) and were then combined into: not at all stressed/somewhat stressed, moderately stressed, and very/extremely stressed.

### 2.4. Statistical analysis

We used SPSS 22.0 for all data analysis. Specifically, first, we used descriptive statistics for the baseline characteristics of the participants and chi-square tests to compare whether there were significant differences in the prevalence of self-reported PMS and depression (including diagnosis of depression and depressive symptoms) in each survey; second, the cross-sectional relationship between self-reported PMS and depression was examined using chi-square tests for diagnosis of depression and frequency of depressive symptoms among women who reported different frequency of PMS symptoms in each survey; third, binary logistic regression and multivariate ordered logistic regression were used to examine the longitudinally predictive effect of self-reported frequency of PMS symptoms in Survey 2 on self-reported diagnosis of depression and frequency of depressive symptoms in each follow-up survey, respectively. In the logistic regression analysis, we added four types of covariates in turn to examine the effect of independent variables on the dependent variable after controlling for each type of covariate. Specifically, Model 1 included only the effects of the independent variable (i.e., self-reported frequency of PMS); Model 2 included the independent variable and demographic variables; Model 3 included all of the above variables and lifestyle habits; Model 4 included all of the above variables and menstrual symptoms; and Model 5 included all of the above variables and other covariates.

Note that in order to better control for the effect of a history of depression, we excluded data from Survey 2 that reported having been diagnosed with depression (i.e., select “yes” for the question “Have you been diagnosed with depression by a doctor in the past 4 years?”); having depressive symptoms (i.e., select “often” for the question “In the past year, have you had depressive symptoms?”); and use of antidepressant medication (i.e., select 1 or more for the question “In the past 4 weeks, how many different types of medication have you used to treat depression?”). In total, 1,199 women were removed due to their history of depression. In addition, 356 women with missing values on these three questions were removed because it could not be determined whether they had a history of depression. Finally, 8,133 validated participants in Survey 2 were remained as baseline. In the subsequent data analysis, all participants without missing values on the dependent variables and covariates were included in the models at each follow-up survey. The specific sample sizes used in each model for each follow-up survey are shown in Supplementary Table 1.

In addition, to assess the robustness of the results, we further examined the effects of missing values in the covariates. The multiple imputation (MI) technique was used to fill in missing values for covariates (including highest educational qualification, marital status, residence, BMI, physical activity, smoking, alcohol consumption, OCPs use, and PSS). Ten completely filled datasets were generated, and then analyzed separately, and the combined results obtained. As MI in SPSS does not yet provide parameter estimates for the combined results (41), we mainly refer to the *p*-values in comparison with the original results.

## 3. Results

### 3.1. Baseline characteristics and prevalence statistics

The sample characteristics of the baseline data are shown in Table 1. Overall, most of the participants were well-educated, unmarried, living in a large city, had a normal weight, had no sleeping difficulty, did not smoke, had no alcohol consumption, had a physical activity habit, did not have irregular menstrual cycles, did not have heavy periods, did not have dysmenorrhea, had no history of abuse, and experienced low levels of stress in 2000.

**Table 1:** Sample characteristics in 2000 (*n* = 9,688).

	***n***	**%**	**Valid %**
**Age** ^a^	9,688		24.1 ± 1.47
**Highest educational qualification**			
Up to high school or equivalent	3,302	34.1	35.4
Trade/diploma	2,268	23.4	24.3
University degree or higher	3,768	38.9	40.4
No response	350	3.6	
**Marital status**			
Married/de facto	4,352	44.9	45.1
Not married/separated/widowed	5,287	54.6	54.9
No response	49	0.5	
**Area of residence**			
Major cities	4,950	51.1	51.3
Inner regional	2,960	30.6	30.7
Outer regional/remote/very remote	1,736	17.9	18.0
No response	42	0.4	
**BMI**			
Underweight (< 18.5)	569	5.9	6.4
Normal weight (18.5 to < 25)	5,554	57.3	62.8
Overweight (25 to < 30)	1,751	18.1	19.8
Obese (≥30)	969	10.0	11.0
No response	845	8.7	
**Sleeping difficulties in the last 12 months**			
Never	5,886	60.8	60.9
Rarely	1,043	10.8	10.8
Sometimes	1,762	18.2	18.2
Often	980	10.1	10.1
No response	17	0.2	
**Physical activity**			
Sedentary	895	9.2	9.5
Low	2,050	31.5	32.3
Moderate	2,208	22.8	23.4
High	3,282	33.9	34.8
No response	252	2.6	
**Smoking**			
Never smoker	5,519	57.0	57.4
Ex-smoker	1,387	14.3	14.4
Current smoker	2,702	27.9	28.1
No response	80	0.8	
**Alcohol consumption**			
Never drinker/rarely drinks	3,659	37.8	38.0
Low risk drinker	5,601	57.8	58.2
Risky/high risk drinker	363	3.7	3.8
No response	65	0.7	
**OCPs use**			
Not using OCPs	4,175	43.1	43.7
Using OCPs	5,368	55.4	56.3
No response	145	1.5	
**Irregular periods**			
Never	6,946	71.7	71.8
Rarely	847	8.7	8.8
Sometimes	1,037	10.7	10.7
Often	841	8.7	8.7
No response	17	0.2	
**Severe period pain**			
Never	6,243	64.4	64.6
Rarely	995	10.3	10.3
Sometimes	1,374	14.2	14.2
Often	1,059	10.9	11.0
No response	17	0.2	
**Heavy periods**			
Never	7,417	76.6	76.7
Rarely	718	7.4	7.4
Sometimes	920	9.5	9.5
Often	616	6.4	6.4
No response	17	0.2	
**History of abuse**			
Yes	3,694	38.1	38.5
No	5,910	61.0	61.5
No response	84	0.9	
**Perceived stress**			
Not at all/somewhat stressed	5,508	56.8	57.2
Moderately stressed	3,515	36.3	36.5
Very/extremely stressed	612	6.2	6.4
No response	55	0.6	
**Medication for depression**			
Yes	427	4.4	4.5
No	8,988	92.8	95.5
No response	273	2.8	

a Mean ± SD.

The prevalence of self-reported PMS symptoms, diagnosis of depression, and depressive symptoms from Surveys 2–8 are shown in Supplementary Table 2. The chi-square test showed significant differences in the proportion of reporting a diagnosis of depression by year [$\chi_{\left(6\right)}^{2}$ = 215.69, *p* < 0.001], with the highest proportion in 2009 (17.7%) being significantly higher than all other years; the proportion in 2018 (15.6%) was significantly higher than that in 2000 (11.6%), 2003 (12.6%), and 2006 (13.5%); and the proportion in 2006 significantly higher than that in 2000 (Bonferroni-corrected *p* < 0.05).

The proportion of self-reported frequency of depressive symptoms also differed significantly by year [$\chi_{\left(18\right)}^{2}$ = 1,033.76, *p* < 0.001]; significantly higher proportions in 2015 (46.1%) and 2018 (48.6%) than in other years; and significantly higher proportions in 2012 (42.2%) than in 2000 (30.9%), 2003 (35.7%), 2006 (35.5%), and 2009 (36.2%); the proportions in 2003, 2006, and 2009 were also higher than in 2000 (Bonferroni-corrected *p* < 0.05).

The proportion of self-reported frequency of PMS symptoms also differed significantly by year [$\chi_{\left(18\right)}^{2}$ = 1,836.52, *p* < 0.001], with significantly higher proportions in 2015 (64.8%) and 2018 (62.7%) than in other years, and significantly higher proportions in 2012 (61.5%) than in 2000 (41.9%), 2003 (48.0%), 2006 (52.9%), and 2009 (50.8%), with significantly higher proportions in 2006 and 2009 than in 2000 and 2003, and significantly higher proportion in 2003 than in 2000 (Bonferroni-corrected *p* < 0.05).

### 3.2. Cross-sectional correlation between PMS and depression

The chi-square test showed that for each survey, as the self-reported frequency of PMS increased, the proportion of individuals who reported being diagnosed with depression increased, and the self-reported frequency of experiencing depressive symptoms also increased (see Table 2).

**Table 2:** The cross-sectional association between PMS and depression.

**Year**	**Variables**	**PMS**				**χ^2^**	***p***	***Post-hoc* tests^a^**
		**Never^1^**	**Rarely^2^**	**Sometimes^3^**	**Often^4^**			
2000	**Diagnosis of depression in last 4 years**					85.99	<0.001	
	Yes	554	70	259	231			4 > 3 > 2 =1
	No	5,001	773	1,662	1,026			1 = 2 > 3 > 4
	**Depressive symptoms in last 12 months**					1,571.68	<0.001	
	Never	4,651	429	1,059	540			1 > 2 = 3 > 4
	Rarely	249	263	304	198			2 > 3 = 4 > 1
	Sometimes	454	109	432	330			4 = 3 > 2 > 1
	Often	267	47	136	203			4 > 3 > 1 (2 =1, 2 = 3)
2003	**Diagnosis of depression in last 3 years**					87.14	<0.001	
	Yes	507	145	255	215			4 > 3 = 2 =1
	No	4,122	1,164	1,722	782			1 = 2 = 3 > 4
	**Depressive symptoms in last 12 months**					941.88	<0.001	
	Never	3,629	791	1,010	389			1 > 2 > 3 > 4
	Rarely	413	287	459	190			3 > 4 > 1 (2 = 3, 2 = 4)
	Sometimes	449	193	402	291			4 > 3 > 2 > 1
	Often	221	56	130	146			4 > 3 > 2 = 1
2006	**Diagnosis of depression in last 3 years**					130.11	<0.001	
	Yes	426	169	320	234			4 > 3 > 2 =1
	No	3,573	1,344	1,691	762			1 = 2 >3 > 4
	**Depressive symptoms in last 12 months**					922.78	<0.001	
	Never	3,311	1,021	1,102	381			1 > 2 > 3 > 4
	Rarely	364	331	430	225			2 = 3 = 4 > 1
	Sometimes	389	204	427	265			4 > 3 > 2 > 1
	Often	181	62	159	157			4 > 3 > 2 = 1
2009	**Diagnosis of depression in last 3 years**					1,07.81	<0.001	
	Yes	564	189	319	236			4 > 3 > 2 =1
	No	3,088	1,119	1,398	545			1 = 2 > 3 > 4
	**Depressive symptoms in last 12 months**					743.67	<0.001	
	Never	3,031	857	959	299			1 > 2 > 3 > 4
	Rarely	352	298	393	184			2 = 3 = 4 > 1
	Sometimes	380	193	350	212			4 > 3 > 2 > 1
	Often	202	56	147	126			4 > 3 > 2 = 1
2012	**Diagnosis of depression in last 3 years**					152.65	<0.001	
	Yes	392	225	428	281			4 > 3 > 2 =1
	No	2,598	1,437	1,755	696			1 = 2 >3 > 4
	**Depressive symptoms in last 12 months**					646.05	<0.001	
	Never	2,201	992	1,059	339			1 > 2 > 3 > 4
	Rarely	395	376	524	227			2 = 3 = 4 > 1
	Sometimes	309	226	460	287			4 > 3 > 2 > 1
	Often	145	87	168	135			4 > 3 > 2 = 1
2015	**Diagnosis of depression in last 3 years**					110.97	<0.001	
	Yes	333	204	323	247			4 > 3 = 2 =1
	No	2,117	1,375	1,707	661			1 = 2 = 3 > 4
	**Depressive symptoms in last 12 months**					446.40	<0.001	
	Never	1,612	888	956	293			1 > 2 > 3 > 4
	Rarely	401	384	529	238			2 = 3 = 4 > 1
	Sometimes	289	231	402	221			4 > 3 > 2 = 1
	Often	146	75	140	156			4 > 3 > 2 (1 =2, 1 = 3)
2018	**Diagnosis of depression in last 3 years**					64.96	<0.001	
	Yes	356	196	308	192			4 > 3 > 2 (1 = 2, 1 = 3)
	No	2,157	1,354	1,614	580			2 > 3 > 4 (1 = 2, 1 = 3)
	**Depressive symptoms in last 12 months**					427.02	<0.001	
	Never	1,625	845	854	229			1 > 2 > 3 > 4
	Rarely	458	423	566	203			2 = 3 = 4 > 1
	Sometimes	330	234	392	240			4 > 3 > 2 = 1
	Often	165	80	149	113			4 > 3 > 2 (1 =2, 1 = 3)

a Bonferroni corrected.

### 3.3. Longitudinal predictive effect of PMS on diagnosis of depression

After controlling for covariates, self-reported frequency of PMS symptoms in 2000 predicted subsequent self-reported diagnosis of depression in most follow-up surveys (see Table 3). Specifically, compared to women who reported “never” had PMS symptoms, those who reported “often” had PMS symptoms in 2000 were more likely to report being diagnosed with depression later [OR_2006_ = 1.72 (1.27–2.32); OR_2012_ = 1.88 (1.44–2.44); OR_2015_ = 1.49 (1.11–1.99); and OR_2018_ = 1.90 (1.41–2.99)], and those women who reported “sometimes” had PMS symptoms in 2000 were more likely to report being diagnosed with depression in 2012 [OR = 1.37 (1.09–1.72)] and 2018 [OR = 1.59 (1.23– 2.05)]. In addition, several covariates influenced the self-reported diagnosis of depression, for example, college and higher-level educational qualifications could decrease the risk of subsequent self-reported diagnosis with depression; while being overweight, having sleeping difficulties, smoking, having a history of abuse, and experiencing stress could increase the risk of subsequent self-reported diagnosis of depression (see Supplementary Table 3).

**Table 3:** Adjusted and unadjusted models for the association between self-reported frequency of PMS symptoms in 2000 and self-reported diagnosis of depression at each follow-up survey.

**Year**	**Premenstrual tension in 2000**	**Model 1^a^**	**Model 2^b^**	**Model 3^c^**	**Model 4^d^**	**Model 5^e^**

		**OR [95% CI]**	**OR [95% CI]**	**OR [95% CI]**	**OR [95% CI]**	**OR [95% CI]**
2003	Never	Reference	Reference	Reference	Reference	Reference
	Rarely	0.99 [0.68–1.43]	0.99 [0.66–1.47]	0.80 [0.53–1.23]	0.84 [0.54–1.31]	0.86 [0.55–1.34]
	Sometimes	**1.42 [1.12–1.81]**	**1.42 [1.10–1.83]**	1.17 [0.89–1.54]	1.173 [0.85–1.52]	1.11 [0.83–1.49]
	Often	**2.06 [1.59–2.66]**	**2.14 [1.63–2.80]**	**1.59 [1.19–2.13]**	**1.43 [1.03–1.98]**	1.26 [0.91–1.76]
2006	Never	Reference	Reference	Reference	Reference	Reference
	Rarely	1.14 [0.82–1.56]	1.35 [0.97–1.89]	1.25 [0.88–1.79]	1.30 [0.90–1.89]	1.32 [0.91–1.92]
	Sometimes	**1.48 [1.19–1.84]**	**1.53 [1.21–1.94]**	1.32 [1.02–1.70]	1.28 [0.98–1.67]	1.28 [0.98–1.68]
	Often	**2.04 [1.62–2.58]**	**2.35 [1.83–3.01]**	**1.91 [1.46–2.50]**	**1.84 [1.37–2.48]**	**1.72 [1.27–2.32]**
2009	Never	Reference	Reference	Reference	Reference	Reference
	Rarely	0.78 [0.56–1.07]	0.82 [0.58–1.16]	0.76 [0.53–1.09]	0.77 [0.53–1.13]	0.77 [0.52–1.13]
	Sometimes	**1.29 [1.06–1.57]**	**1.34 [1.08–1.65]**	1.19 [0.95–1.49]	1.18 [0.93–1.50]	1.17 [0.92–1.48]
	Often	**1.54 [1.22–1.93]**	**1.67 [1.31–2.12]**	**1.44 [1.12–1.86]**	**1.40 [1.05–1.86]**	1.31 [0.98–1.74]
2012	Never	Reference	Reference	Reference	Reference	Reference
	Rarely	0.96 [0.71–1.29]	0.96 [0.70–1.32]	0.92 [0.66–1.28]	0.89 [0.63–1.27]	0.90 [0.63–1.27]
	Sometimes	**1.45 [1.20–1.76]**	**1.55 [1.27–1.90]**	**1.45 [1.17–1.80]**	**1.39 [1.11–1.74]**	**1.37 [1.09–1.72]**
	Often	**2.19 [1.78–2.70]**	**2.41 [1.93–3.00]**	**2.16 [1.71–2.73]**	**2.03 [1.57–2.64]**	**1.88 [1.44–2.44]**
2015	Never	Reference	Reference	Reference	Reference	Reference
	Rarely	0.97 [0.71–1.32]	1.04 [0.75–1.45]	1.11 [0.79–1.56]	1.02 [0.71–1.46]	1.03 [0.72–1.48]
	Sometimes	**1.28 [1.04–1.58]**	**1.36 [1.10–1.70]**	**1.34 [1.06–1.68]**	**1.26 [0.99–1.61]**	1.26 [0.98–1.61]
	Often	**1.74 [1.38–2.20]**	**1.83 [1.43–2.34]**	**1.69 [1.30–2.19]**	**1.63 [1.22–2.17]**	**1.49 [1.11–1.99]**
2018	Never	Reference	Reference	Reference	Reference	Reference
	Rarely	1.21 [0.89–1.64]	1.30 [0.94–1.80]	1.34 [0.95–1.89]	**1.43 [1.00–2.04]**	1.42 [0.99–2.03]
	Sometimes	**1.39 [1.12–1.73]**	**1.48 [1.18–1.86]**	**1.51 [1.19–1.92]**	**1.59 [1.24–2.05]**	**1.59 [1.23–2.05]**
	Often	**1.73 [1.36–2.21]**	**1.92 [1.49–2.48]**	**1.86 [1.42–2.44]**	**2.07 [1.54–2.79]**	**1.90 [1.41–2.56]**

OR, Odds Ratio; CI, confidence interval.

Significant ORs are in bold. The sample sizes used for the analysis of each model are summarized in Supplementary Table 1.

a Model 1 included only the effect of self-reported frequency of PMS.

b Model 2 adjusted for age, highest educational qualification, marital status, area of residence, and BMI.

c Model 3 adjusted for variables in Model 2 plus for sleeping difficulties, physical activity, smoking, alcohol consumption, and OCPs use.

d Model 4 adjusted for variables in Model 3 plus for irregular periods, severe period pain, and heavy periods.

e Model 5 adjusted for variables in Model 4 plus for history of abuse and perceived stress.

### 3.4. Longitudinal predictive effect of PMS on depressive symptoms

After controlling for covariates, self-reported frequency of PMS symptoms in 2000 predicted self-reported frequency of depressive symptoms in each follow-up survey (see Table 4). Specifically, compared to women who reported “never” had PMS symptoms, those who reported “sometimes” had PMS symptoms in 2000 reported more frequently depressive symptoms later [OR_2003_ = 1.34 (1.14–1.57); OR_2006_ = 1.26 (1.07–1.48); OR_2009_ = 1.25 (1.06–1.47); OR_2012_ = 1.42 (1.21–1.65); OR_2015_ = 1.34 (1.14–1.58); and OR_2018_ = 1.25 (1.06–1.47)], and those individuals who reported “often” had PMS symptoms in 2000 also reported more frequent depressive symptoms later [OR_2003_ = 2.07 (1.72–2.50); OR_2006_ = 1.88 (1.56–2.28); OR_2009_ = 1.71 (1.40–2.08); OR_2012_ = 1.82 (1.50–2.20); OR_2015_ = 1.77 (1.45–2.17); and OR_2018_ = 1.60 (1.31–1.96)]. Similarly, some covariates also influenced the self-reported frequency of depressive symptoms (see Supplementary Table 4).

**Table 4:** Adjusted and unadjusted models for the association between self-reported frequency of PMS symptoms in 2000 and self-reported frequency of depressive symptoms at each follow-up survey.

**Year**	**PMS in 2000**	**Model 1^a^**	**Model 2^b^**	**Model 3^c^**	**Model 4^d^**	**Model 5^e^**
		**OR [95% CI]**	**OR [95% CI]**	**OR [95% CI]**	**OR [95% CI]**	**OR [95% CI]**
2003	Never	Reference	Reference	Reference	Reference	Reference
	Rarely	**1.28 [1.06–1.54]**	**1.25 [1.02–1.52]**	1.04 [0.84–1.29]	1.04 [0.83–1.30]	1.05 [0.84–1.32]
	Sometimes	**1.66 [1.45–1.89]**	**1.67 [1.45–1.92]**	**1.41 [1.21–1.63]**	**1.37 [1.17–1.60]**	**1.34 [1.14–1.57]**
	Often	**2.75 [2.37–3.19]**	**2.95 [2.52–3.45]**	**2.35 [1.99–2.78]**	**2.29 [1.90–2.76]**	**2.07 [1.72–2.50]**
2006	Never	Reference	Reference	Reference	Reference	Reference
	Rarely	1.18 [0.98–1.43]	1.21 [0.99–1.48]	0.72 [0.84–1.29]	1.04 [0.83–1.30]	1.05 [0.84–1.32]
	Sometimes	**1.55 [1.35–1.76]**	**1.59 [1.38–1.83]**	**1.34 [1.15–1.56]**	**1.27 [1.08–1.49]**	**1.26 [1.07–1.48]**
	Often	**2.48 [2.13–2.88]**	**2.78 [2.37–3.26]**	**2.20 [1.85–2.61]**	**2.07 [1.71–2.49]**	**1.88 [1.56–2.28]**
2009	Never	Reference	Reference	Reference	Reference	Reference
	Rarely	1.08 [0.89–1.32]	1.11 [0.90–1.38]	0.97 [0.78–1.22]	0.94 [0.74–1.19]	0.95 [0.75–1.20]
	Sometimes	**1.50 [1.31–1.72]**	**1.54 [1.34–1.78]**	**1.29 [1.11–1.51]**	**1.29 [1.10–1.52]**	**1.25 [1.06–1.47]**
	Often	**2.11 [1.80–2.48]**	**2.33 [1.97–2.75]**	**1.86 [1.56–2.22]**	**1.85 [1.52–2.25]**	**1.71 [1.40–2.08]**
2012	Never	Reference	Reference	Reference	Reference	Reference
	Rarely	1.11 [0.92–1.34]	1.14 [0.94–1.40]	1.06 [0.86–1.30]	1.02 [0.82–1.28]	1.04 [0.84–1.30]
	Sometimes	**1.54 [1.35–1.76]**	**1.66 [1.45–1.91]**	**1.51 [1.30–1.75]**	**1.45 [1.24–1.69]**	**1.42 [1.21–1.65]**
	Often	**2.20 [1.88–2.57]**	**2.49 [2.12–2.93]**	**2.13 [1.79–2.53]**	**2.01 [1.66–2.42]**	**1.82 [1.50–2.20]**
2015	Never	Reference	Reference	Reference	Reference	Reference
	Rarely	1.11 [0.92–1.35]	1.15 [0.94–1.41]	1.08 [0.87–1.34]	1.09 [0.87–1.37]	1.11 [0.88–1.40]
	Sometimes	**1.42 [1.24–1.63]**	**1.49 [1.29–1.72]**	**1.39 [1.19–1.62]**	**1.35 [1.15–1.58]**	**1.34 [1.14–1.58]**
	Often	**2.18 [1.85–2.56]**	**2.37 [2.00–2.81]**	**2.02 [1.68–2.42]**	**1.95 [1.60–2.38]**	**1.77 [1.45–2.17]**
2018	Never	Reference	Reference	Reference	Reference	Reference
	Rarely	1.00 [0.83–1.22]	1.02 [0.83–1.26]	0.94 [0.75–1.17]	0.98 [0.78–1.23]	0.99 [0.79–1.25]
	Sometimes	**1.40 [1.22–1.60]**	**1.45 [1.25–1.67]**	**1.31 [1.12–1.52]**	**1.28 [1.09–1.51]**	**1.25 [1.06–1.47]**
	Often	**1.99 [1.70–2.34]**	**2.15 [1.82–2.55]**	**1.84 [1.54–2.20]**	**1.78 [1.46–2.16]**	**1.60 [1.31–1.96]**

OR, Odds Ratio; CI, confidence interval.

Significant ORs are in bold. The sample sizes used for the analysis of each model are summarized in Supplementary material.

a Model 1 included only the effect of self-reported frequency of PMS.

b Model 2 adjusted for age, highest educational qualification, marital status, area of residence, and BMI.

c Model 3 adjusted for variables in Model 2 plus for sleeping difficulties, physical activity, smoking, alcohol consumption, and OCPs use.

d Model 4 adjusted for variables in Model 3 plus for irregular periods, severe period pain, and heavy periods.

e Model 5 adjusted for variables in Model 4 plus for history of abuse and perceived stress.

### 3.5. Sensitivity analysis

The results of multiple imputation (see Supplementary Tables 5, 6) showed that the results were the same as those with missing values, except that, compared to women who reported “never” had PMS symptoms in 2000, those women who reported “sometimes” had PMS symptoms were significantly more likely to report being diagnosed with depression in 2006.

## 4. Discussion

The present study examined the predictive role of self-reported frequency of PMS symptoms on depression using longitudinal cohort data from Australia, and showed that self-reported PMS symptoms at the baseline predicted subsequent self-reported diagnosis of depression and frequency of depressive symptoms over a developmental period of up to 18 years in the reproductive stage. We were able to capture the self-reported prevalence of PMS and depression, and their changes, over a longer period of time, as well as the long-term predictive role of self-reported frequency of PMS for depression.

This study found that the proportion of women who reported being diagnosed with depression peaked in 2009 (31–36 years old), then decreased slightly, but showed an overall increasing trend. Most previous studies have roughly categorized participants into several age groups, such as 20–39, 40–59, and 60+ (42); or selected only a representative number of age groups, such as comparing differences in the prevalence of depression between 20–24, 40–44, and 60–64-year-olds (43), while the change trends between the ages of 22–45 were not analyzed. The cohort data used in this study enabled us to compare the prevalence of self-reported depression between the ages of 22–45 years with a 3-year sampling interval, and found the highest prevalence occurred between the ages of 31–36 years, which is largely consistent with the findings of Weinberger et al. (44). Their study conducted annual statistics on the prevalence of depression for participants aged 12–17, 18–25, 26–34, 35–49, and 50+ years old during the ten-year period from 2005 to 2015, and found that for the groups aged 12–17, 18–25, and 50+ years old, the prevalence of depression showed a significant upward trend over the 10-year period, which means that the prevalence of depression was increasing between the ages of 12–35, but was relatively stable after the age of 36 (because there was no significant increase in the prevalence of depression among the 35–49 age group). This may be due to the fact that 31–35 years is a transitional period, with women facing various stressors such as work, marriage, and childbirth, but lacking relatively effective coping strategies; whereas after the age of 36, their life status is relatively stable and they have found more mature coping strategies in the face of familiar stressors (45, 46). In addition, the self-reported frequency of depressive and PMS symptoms maintained an increasing trend until reaching a maximum in 2015 and 2018, similar to previous studies with samples from other countries (47–49). This indicated that between the ages of 22–40, women's PMS symptoms and depressive symptoms gradually increased, but remained relatively stable after age 40 (as the difference between 2015 and 2018 was not significant); it also added evidence for symptom frequency to results from previous studies addressing symptom severity.

Results addressing the cross-sectional relationship between self-reported PMS and depression found that as women's self-reported frequency of PMS increased, so did the self-reported frequency of diagnosis of depression and experience of depressive symptoms, in line with previous small sample studies (23, 26, 28). More importantly, we found that baseline self-reported frequency of PMS symptoms predicted subsequent self-reported diagnosis of depression and depressive symptoms. Halbreich (50) first suggested that PMS might be a risk factor for depression, and the results of the present study provide empirical support for this from both cross-sectional and longitudinal data. In addition, risk factors for depression change over time (51), meaning their predictive effect for depression may be explained in more than one way: on the one hand, worsening risk-factor levels may lead to the onset of depression, i.e., the risk-escalation hypothesis (52); on the other hand, the cumulative effect of risk-factor levels may lead to the occurrence of depression, i.e., the chronic-risk hypothesis (53). A recent study by Mu et al. (54) examined 16 risk factors for depression and consistently found that it was the average level of risk factors, rather than abrupt changes, that led to the first onset of depression, supporting the chronic-risk hypothesis. Our findings, using PMS as a risk factor, support the chronic-risk hypothesis, meaning that the predictive effect of self-reported frequency of PMS for depression is long-term and exhibits a greater and more stable long-term effect on the prediction of diagnosis of depression.

Two possible pathways may exist for the predictive role of self-reported frequency of PMS on depression. On the one hand, according to the windows of sensitivity theory (12), sharp changes in progesterone levels during the luteal phase lead to changes in brain networks, and this further leads women to develop a memory bias for negative events during the luteal phase; this results in an increase in negative emotions and susceptibility to mood disorders. Evidence regarding the relationship between hormonal fluctuations and emotional symptoms has also been obtained from menopause. Specifically, numerous pieces of evidence suggest that menopause, with its dramatic hormonal fluctuations, is strongly associated with new onset of depressed mood (55) and increased risk of greater depressive symptoms (56) among women with no history of depression. In addition, compared with women who used hormonal interventions to ease the symptoms associated with perimenopause, those without using hormonal interventions were more likely to experience a severe depressive episode (57). Women with PMS are particularly sensitive to hormonal fluctuations (58) and brain networks change more drastically during the menstrual cycle. As a result, women with PMS reported more emotional symptoms during the luteal phase of the menstrual cycle and greater sensitivity to depressive symptoms than women without PMS. In other words, hormonal changes and fluctuations were associated with an increased risk of depressive symptoms (59), especially for women with PMS. On the other hand, women with PMS experience a range of physical, psychological, and behavioral symptoms during the luteal phase of menstrual cycle, which leads them to develop a negative attitude toward menstruation (60). Menstruation becomes a chronic stressor for them (29), and according to the theory of allostatic load, when individuals are regularly exposed to chronic stress, their physiological responses may be dysregulated, resulting in insufficient resources to cope with other stressors (61). Previous empirical studies have also found abnormal stress responses in women with PMS in the premenstrual period (62, 63), suggesting this may create a vicious cycle that makes women with PMS experience more emotional symptoms, and put them at a higher risk of developing depression. However, based on the results of this study, it is uncertain which pathway plays a more important role in the prediction of depression by PMS, or whether they are equal. Future studies should further examine the longitudinal predictive mechanisms of PMS for depression.

It is important to note that our results found that the predictive effect of self-reported frequency of PMS symptoms on depressive symptoms was more stable and sensitive than the predictive effect on diagnosis of depression, as evidenced by the significant predictive effect for each follow-up survey and that women who reported “sometimes” had PMS symptoms in 2000 reported significantly more subsequent depressive symptoms than those reported “never” had PMS symptoms in 2000. This may be due to the difference between the two measures. First, participants may have some stigma about being diagnosed with depression (64), leading to less truthful reporting by some participants (65). However, the stigma of depression decreases over time (66), which may have resulted in participants' subsequent reports on diagnosis of depression being more truthful, while reports of depressive symptoms were less affected. Second, the variables involved in this study were retrospective subjective reports, which may be subject to recall bias (67). Participants reported on diagnosis of depression for the past 3 years, while they reported depressive symptoms for the past 12 months. The relatively shorter period of recall time may have made recall easier, and the report closer to the truth. The effect of the mood state measurement pattern on the results has been confirmed in previous studies (68), and the question of whether the difference in the predictive effect of the data reported for both variables was caused by the difference in the measurement pattern can be ruled out by comparing the response times in the future. Finally, the question about diagnosis of depression required participants to make a binary choice, whereas the questions about depressive symptoms required participants to choose among different frequencies of symptoms; the way depressive symptoms were reported was more consistent with the way PMS symptoms were reported, which may also have led to a more stable predictive effect on depressive symptoms. In conclusion, our findings suggest a more stable and sensitive predictive effect of self-reported frequency of PMS symptoms on self-reported frequency of depressive symptoms, but a more complex predictive effect of self-reported frequency of PMS symptoms on self-reported depressive diagnoses. Furthermore, as with the measurement of PMS symptoms, the diagnosis of depression and depressive symptoms were also measured by a simple single question rather than rating scales or specific questionnaires, this may cause the common method bias problem thus making the relationship between PMS and depression stronger than it really is. Therefore, conclusions should be drawn with caution.

In addition, this study found that covariates such as highest educational qualification, sleeping difficulties, perceived stress, history of abuse, smoking, and physical activity also played a predictive role for self-reported diagnosis of depression and depressive symptoms, similar to previous studies (22, 24, 40, 69, 70). This study examined the long-term predictive effect of self-reported frequency of PMS symptoms on self-reported depression for the first time using longitudinal cohort data and collected a large number of demographic variables and other covariates, which allowed us to control the sampling bias, and examine the predictive effect of self-reported frequency of PMS symptoms on depression after excluding the interference of other factors. Robustness analysis also revealed no effect of missing values on our findings, meaning that the results had high stability. This provides evidence for our use of PMS as a risk factor that may be used for early identification and screening for depression and allows for generalization of the findings to Australian women of the same age group.

However, the present study still has some limitations, which focused on the following aspects. First and foremost, it is inaccurate to measure PMS using a single question of the retrospective nature, which poses the following problems. Firstly, because retrospective self-reported PMS measurements were more sensitive to mood disorders and cycle phases than prospective measurements with daily symptoms calendars (71), it is not equivalent to a real PMS diagnosis *via* prospective measurement (72), which does make the stability of the study results very challenging. Secondly, a single question asking participants about the frequency of PMS prevented us from examining the severity of symptoms and which symptoms of PMS were predictive of depression. Last but not least, “premenstrual tension” is an old term from the International Statistical Classification of Diseases and Related Health Problems, Tenth Edition (ICD-10), which refers to any premenstrual symptoms such as tension or migraine without consideration of the type or severity of the symptoms (73). This definition serves as the most relaxed criteria of PMS and differs from the definition of PMS/PMDD by Diagnostic and Statistical Manual of Mental Disorders Fifth Edition (DSM-V) (25) or the definition of premenstrual disorders by the International Society of Premenstrual Disorders (ISPMD) (74). There are extenuating circumstances for using this measure, such as (1) this is a cohort dataset with a very large amount of data (including demographic variables, health behaviors, diagnoses, symptoms, general measures of health, access to and use of a range of health services, contraception and reproduction, childbirth and parenting, experiences of intimate partner violence, and work-life balance, a total of more than 500 questions) collected, single questions could save sampling time, (2) it was collected as early as in 2000 when premenstrual tension was widely used and studied, (3) the object of the study is not a clinical sample. Given the acceptance of this measure in previous studies [e.g., (38, 39)], we also used the term “premenstrual syndrome” in this paper in order to maintain consistency with other studies. However, there might be a slight difference between the definition of premenstrual tension at that time and the recent definitions of PMS, so the conclusion obtained should be carefully considered and validated in the future using recent standardized measurements. In addition, because we did not measure depression-related data in Survey 1, we know nothing about participants' history of depression before Survey 2, which may cause some confusion in the results. Finally, because we had a high attrition rate, the remaining individuals may have been those that were more concerned with health issues, or that had some health issues of their own; as such, the results obtained may have been somewhat overestimated in terms of prevalence (40).

## 5. Conclusion and implications

The longitudinal predictive relationship of the self-reported frequency of PMS symptoms in their early twenties on the self-reported frequency of depressive symptoms and the self-reported diagnosis of depression in subsequent decades was initially confirmed in the ALSWH 1973-78 cohort. Therefore, early intervention for PMS (e.g., hormonal interventions or cognitive behavioral therapy) may not only reduce the physical and psychological distress of women with PMS during the luteal phase of the menstrual cycle [e.g., (75, 76)], but also reduce their risk of being diagnosed with depression later in life. Given that current intervention studies on depressive symptoms in women with PMS are cross-sectional studies [e.g., (77)], future longitudinal studies should be conducted to provide further confirmation of this issue by comparing differences in subsequent presence and severity of depressive symptoms between women who sought treatment for PMS and those who did not receive treatment.

## Data availability statement

ALSWH survey data are owned by the Australian Government Department of Health and due to the personal nature of the data collected, release by ALSWH is subject to strict contractual and ethical restrictions. Ethical review of ALSWH is by the Human Research Ethics Committees at the University of Queensland and the University of Newcastle. De-identified data are available to collaborating researchers where a formal request to make use of the material has been approved by the ALSWH Data Access Committee. The committee is receptive of requests for datasets required to replicate results. Requests to access the datasets should be directed to https://alswh.org.au/for-data-users/applying-for-data/.

## Ethics statement

The studies involving human participants were reviewed and approved by the Human Research Ethics Committees (HRECs) of the Universities of Newcastle and Queensland (approval number H076-0795). The patients/participants provided their written informed consent to participate in this study.

## Author contributions

Material preparation, data collection, and analysis were performed by LH and LC. The first draft of the manuscript was written by LH. All authors contributed to the study conception, design, commented on previous versions of the manuscript, and read and approved the final manuscript.

## References

[1] Lacey BWH. Global age-sex-specific fertility, mortality, healthy life expectancy (HALE), and population estimates in 204 countries and territories, 1950–2019: A comprehensive demographic analysis for the Global Burden of Disease Study 2019. Lancet. (2020) 396:1160–203. 10.1016/s0140-6736(20)30977-6 33069325 PMC7566045

[2] Vos T Lim SS Abbafati C Abbas KM Abbasi M Abbasifard M. Global burden of 369 diseases and injuries in 204 countries and territories, 1990–2019: A systematic analysis for the Global Burden of Disease Study 2019. Lancet. (2020) 396:1204–22. 10.1016/S0140-6736(20)30925-9 33069326 PMC7567026

[3] Lim GY Tam WW Lu Y Ho CS Zhang MW Ho RC. Prevalence of depression in the community from 30 countries between 1994 and 2014. Sci Rep. (2018) 8:1–10. 10.1038/s41598-018-21243-x 29434331 PMC5809481

[4] Chi I Yip PSF Chiu HFK Chou KL Chan KS Kwan CW. Prevalence of depression and its correlates in Hong Kong's Chinese older adults. Am J Geriatr Psychiatry. (2005) 13:409–16. 10.1097/00019442-200505000-00010 15879590

[5] Kessler RC McGonagle KA Swartz M Blazer DG Nelson CB. Sex and depression in the National Comorbidity Survey I: Lifetime prevalence, chronicity and recurrence. J Affect Disord. (1993) 29:85–96. 10.1016/0165-0327(93)90026-G 8300981

[6] Pachana NA McLaughlin D Leung J Byrne G Dobson A. Anxiety and depression in adults in their eighties: Do gender differences remain? Int Psychogeriatr. (2012) 24:145–50. 10.1017/S1041610211001372 21777505

[7] Cyranowski JM Frank E Young E Shear MK. Adolescent onset of the gender difference in lifetime rates of major depression: A theoretical model. Arch Gen Psychiatry. (2000) 57:21–7. 10.1001/archpsyc.57.1.21 10632229

[8] Twenge JM Nolen-Hoeksema S. Age, gender, race, socioeconomic status, and birth cohort difference on the children's depression inventory: A meta-analysis. J Abnorm Psychol. (2002) 111:578. 10.1037/0021-843X.111.4.578 12428771

[9] Girgus JS Yang K. Gender and depression. Curr Opin Psychol. (2015) 4:53–60. 10.1016/j.copsyc.2015.01.019

[10] Hyde JS Mezulis AH Abramson LY. The ABCs of depression: Integrating affective, biological, and cognitive models to explain the emergence of the gender difference in depression. Psychol Rev. (2008) 115:291–313. 10.1037/0033-295X.115.2.291 18426291

[11] Schiller CE Johnson SL Abate AC Schmidt PJ Rubinow DR. Reproductive steroid regulation of mood and behavior. Compr Physiol. (2016) 6:1135. 10.1002/cphy.c150014 27347888 PMC6309888

[12] Andreano JM Touroutoglou A Dickerson B Barrett LF. Hormonal cycles, brain network connectivity, and windows of vulnerability to affective disorder. Trends Neurosci. (2018) 41:660–76. 10.1016/j.tins.2018.08.007 30274602 PMC6481680

[13] Eley TC Sugden K Corsico A Gregory AM Sham P McGuffin P. Gene–environment interaction analysis of serotonin system markers with adolescent depression. Mol Psychiatry. (2004) 9:908–15. 10.1038/sj.mp.4001546 15241435

[14] Casper RF Crowley WF Jr. Patient Education: Premenstrual syndrome (PMS) Premenstrual Dysphoric Disorder (PMDD) (Beyond the Basics). (2019). Available online at: https://medilib.ir/uptodate/show/2159 (accessed February 24, 2021).

[15] Hou L Zhou R. Patterns of premenstrual syndrome and depression symptoms in Chinese female university students: Results of a latent profile analysis. J Affect Disord. (2021) 293:64–70. 10.1016/j.jad.2021.06.017 34174472

[16] Afifi TO Brownridge DA Cox BJ Sareen J. Physical punishment, childhood abuse and psychiatric disorders. Child Abuse Neglect. (2006) 30:1093–103. 10.1016/j.chiabu.2006.04.006 17010436

[17] Anastasiades MH Kapoor S Wootten J Lamis DA. Perceived stress, depressive symptoms, and suicidal ideation in undergraduate women with varying levels of mindfulness. Arch Womens Ment Health. (2017) 20:129–38. 10.1007/s00737-016-0686-5 27778150

[18] Bertone-Johnson ER Whitcomb BW Missmer SA Manson JE Hankinson SE Rich-Edwards JW. Early life emotional, physical, and sexual abuse and the development of premenstrual syndrome: A longitudinal study. J Womens Health. (2014) 23:729–39. 10.1089/jwh.2013.4674 25098348 PMC4158950

[19] Choi SH Hamidovic A. Association between smoking and premenstrual syndrome: A meta-analysis. Front Psychiatry. (2020) 11:575526. 10.3389/fpsyt.2020.575526 33324253 PMC7725748

[20] del Mar Fernández M Regueira-Méndez C Takkouche B. Psychological factors and premenstrual syndrome: A Spanish case-control study. PLoS ONE. (2019) 14:e0212557. 10.1371/journal.pone.0212557 30840651 PMC6402625

[21] Ito K Doi S Isumi A Fujiwara T. Association between childhood maltreatment history and premenstrual syndrome. Int J Env Res Pub Hea. (2021) 18:781. 10.3390/ijerph18020781 33477613 PMC7831299

[22] Li D Chu CM Lai V. A developmental perspective on the relationship between child sexual abuse and depression: A systematic and meta-analytic review. Child Abuse Rev. (2020) 29:27–47. 10.1002/car.2592

[23] Padhy SK Sarkar S Beherre PB Rathi R Panigrahi M Patil PS. Relationship of premenstrual syndrome and premenstrual dysphoric disorder with major depression: Relevance to clinical practice. Indian J Psychol Med. (2015) 37:159–64. 10.4103/0253-7176.155614 25969600 PMC4418247

[24] Wootton RE Richmond RC Stuijfzand BG Lawn RB Sallis HM Taylor GMJ. Evidence for causal effects of lifetime smoking on risk for depression and schizophrenia: A Mendelian randomisation study. Psychol Med. (2020) 50:2435–43. 10.1017/S0033291719002678 31689377 PMC7610182

[25] American Psychiatric Association. Diagnostic and Statistical Manual of Mental Disorders. 5th ed. Washington, DC: American Psychiatric Association. (2013). 10.1176/appi.books.9780890425596

[26] Yonkers KA Pearlstein T Rosenheck RA. Premenstrual disorders: Bridging research and clinical reality. Arch Womens Ment Health. (2003) 6:287–92. 10.1007/s00737-003-0026-4 14628181

[27] Forrester-Knauss C Zemp Stutz E Weiss C Tschudin S. The interrelation between premenstrual syndrome and major depression: Results from a population-based sample. BMC Public Health. (2011) 11:1–11. 10.1186/1471-2458-11-795 21992230 PMC3209462

[28] Sadr SS Ardestani SMS Razjouyan K Daneshvari M Zahed G. Premenstrual syndrome and comorbid depression among medical students in the internship stage: A descriptive study. Iran J Psychiatry Behav Sci. (2014) 8:74–9. 25798178 PMC4364481

[29] Breaux C Hartlage S Gehlert S. Relationships of premenstrual dysphoric disorder to major depression and anxiety disorders: A re-examination. J Psychosom Obst Gyn. (2000) 21:17–24. 10.3109/01674820009075604 10907211

[30] Graze KK Nee J Endicott J. Premenstrual depression predicts future major depressive disorder. Acta Psychiatr Scand. (1990) 81:201–5. 10.1111/j.1600-0447.1990.tb06479.x 2327284

[31] Hartlage SA Arduino KE Gehlert S. Premenstrual dysphoric disorder and risk for major depressive disorder: A preliminary study. J Clin Psychol. (2001) 57:1571–8. 10.1002/jclp.1119 11745598

[32] Guloksuz S van Os J. The slow death of the concept of schizophrenia and the painful birth of the psychosis spectrum. Psychol Med. (2018) 48:229–44. 10.1017/S0033291717001775 28689498

[33] Hankin BL Fraley RC Lahey BB Waldman ID. Is depression best viewed as a continuum or discrete category? A taxometric analysis of childhood and adolescent depression in a population-based sample. J Abnorm Psychol. (2005) 114:96. 10.1037/0021-843X.114.1.96 15709816

[34] Sun X So SHW Chung LKH Chiu C-D Chan RCK Leung PWL. Longitudinal bifactor modeling of anxiety, depression and schizotypy-The role of rumination as a shared mechanism. Schizophr Res. (2022) 240:153–61. 10.1016/j.schres.2022.01.005 35030443

[35] Lee C Dobson AJ Brown WJ Bryson L Byles J Warner-Smith P. Cohort profile: The Australian longitudinal study on women's health. Int J Epidemiol. (2005) 34:987–91. 10.1093/ije/dyi098 15894591

[36] Dobson AJ Hockey R Brown WJ Byles JE Loxton DJ McLaughlin D. Cohort profile update: Australian longitudinal study on women's health. Int J Epidemiol. (2015) 44:1547–1547f. 10.1093/ije/dyv110 26130741

[37] Bell S Lee C. Development of the perceived stress questionnaire for young women. Psychol Health Med. (2002) 7:189–201. 10.1080/1354850012011608522477842

[38] Cao S Jones M Tooth L Mishra G. Does premenstrual syndrome before pregnancy increase the risk of postpartum depression? Findings from the Australian Longitudinal Study on Women's Health. J Affect Disord. (2021) 279:143–8. 10.1016/j.jad.2020.09.130 33049432

[39] Cao S Jones M Tooth L Mishra GD. Generational differences in the prevalence of postpartum depression among young Australians: A comparison of two cohorts born 17 years apart. Arch Womens Ment Health. (2022) 25:199–214. 10.1007/s00737-021-01182-9 34528134

[40] Jackson ML Sztendur EM Diamond NT Byles JE Bruck D. Sleep difficulties and the development of depression and anxiety: A longitudinal study of young Australian women. Arch Womens Ment Health. (2014) 17:189–98. 10.1007/s00737-014-0417-8 24647705

[41] Van Ginkel JR Linting M Rippe RCA van der Voort A. Rebutting existing misconceptions about multiple imputation as a method for handling missing data. J Pers Assess. (2020) 102:297–308. 10.1080/00223891.2018.1530680 30657714

[42] Brody DJ Pratt LA Hughes JP. Prevalence of Depression among Adults Aged 20 Over: United States, 2013-2016. NCHS Data Brief. 303. (2018). p. 1–8. Available online at: https://www.cdc.gov/nchs/products/databriefs/db303.htm (accessed February 13, 2018). 29638213

[43] Batterham PJ Christensen H Mackinnon AJ. Modifiable risk factors predicting major depressive disorder at four year follow-up: A decision tree approach. BMC Psychiatry. (2009) 9:1–8. 10.1186/1471-244X-9-75 19930610 PMC2784764

[44] Weinberger AH Gbedemah M Martinez AM Nash D Galea S Goodwin RD. Trends in depression prevalence in the USA from 2005 to 2015: Widening disparities in vulnerable groups. Psychol Med. (2018) 48:1308–15. 10.1017/S0033291717002781 29021005

[45] Meléndez JC Mayordomo T Sancho P Tomás JM. Coping strategies: Gender differences and development throughout life span. Span J Psychol. (2012) 15:1089–98. 10.5209/rev_SJOP.2012.v15.n3.39399 23156917

[46] Rich JL Byrne JM Curryer C Byles JE Loxton D. Prevalence and correlates of depression among Australian women: A systematic literature review, January 1999-January 2010. BMC Res Notes. (2013) 6:1–16. 10.1186/1756-0500-6-424 24138703 PMC3827921

[47] Freeman EW Rickels K Schweizer E Ting T. Relationships between age and symptom severity among women seeking medical treatment for premenstrual symptoms. Psychol Med. (1995) 25:309–15. 10.1017/S0033291700036205 7675918

[48] Keyes KM Gary D O'Malley PM Hamilton A Schulenberg J. Recent increases in depressive symptoms among US adolescents: Trends from 1991 to 2018. Soc Psych Psych Epid. (2019) 54:987–96. 10.1007/s00127-019-01697-8 30929042 PMC7015269

[49] Mata DA Ramos MA Bansal N Khan R Guille C Di Angelantonio E. Prevalence of depression and depressive symptoms among resident physicians: A systematic review and meta-analysis. J Am Med Assoc. (2015) 314:2373–83. 10.1001/jama.2015.15845 26647259 PMC4866499

[50] Halbreich U. Premenstrual dysphoric disorders, anxiety, and depressions: vulnerability traits or comorbidity. Arch Gen Psychiatry. (1995) 52:606. 10.1001/archpsyc.1995.03950190088013 7598638

[51] Roberts BW Walton KE Viechtbauer W. Patterns of mean-level change in personality traits across the life course: A meta-analysis of longitudinal studies. Psychol Bull. (2006) 132:1. 10.1037/0033-2909.132.1.1 16435954

[52] Laceulle OM Ormel J Vollebergh WAM Van Aken MAG Nederhof E. A test of the vulnerability model: Temperament and temperament change as predictors of future mental disorders–the TRAILS study. J Child Psychol Psyc. (2014) 55:227–36. 10.1111/jcpp.12141 24552482

[53] Klein DN Shankman SA Lewinsohn PM Seeley JR. Subthreshold depressive disorder in adolescents: Predictors of escalation to full-syndrome depressive disorders. J Am Acade Child Psy. (2009) 48:703–10. 10.1097/CHI.0b013e3181a56606 19465876 PMC2866498

[54] Mu W Li K Tian Y Perlman G Michelini G Watson D. Dynamic risk for first onset of depressive disorders in adolescence: Does change matter? Psychol Med. (2021) 2021:1–9. 10.1017/S0033291721004190 34802476

[55] Freeman EW Sammel MD Lin H Nelson DB. Associations of hormones and menopausal status with depressed mood in women with no history of depression. Arch Gen Psychiatry. (2006) 63:375–82. 10.1001/archpsyc.63.4.375 16585466

[56] Mulhall S Andel R Anstey KJ. Variation in symptoms of depression and anxiety in midlife women by menopausal status. Maturitas. (2018) 108:7–12. 10.1016/j.maturitas.2017.11.005 29290217

[57] Cohen LS Soares CN Vitonis AF Otto MW Harlow BL. Risk for new onset of depression during the menopausal transition: The Harvard study of moods and cycles. Arch Gen Psychiatry. (2006) 63:385–90. 10.1001/archpsyc.63.4.385 16585467

[58] Schmidt PJ Nieman LK Danaceau MA Adams LF Rubinow DR. Differential behavioral effects of gonadal steroids in women with and in those without premenstrual syndrome. New Engl J Med. (1998) 338:209–16. 10.1056/NEJM199801223380401 9435325

[59] Callegari C Ielmini M Caselli I Lucca G Isella C Diurni M. Paroxetine vs. vortioxetine for depressive symptoms in postmenopausal transition: A preliminary study. Psychopharmacol Bull. (2019) 49:28. 10.64719/pb.4586 PMC6386432 30858637

[60] Philip SM Suresh A Priyadharshini Dhanasekaran G Sivasankaran PAPR Nagendra VH. Assessment of menstrual attitudes and predictors for premenstrual syndrome in university students of Ooty, South India. Posit Sch Psychol. (2022) 2022:3736–46.

[61] McEwen BS Stellar E. Stress and the individual: Mechanisms leading to disease. Arch Intern Med. (1993) 153:2093–101. 10.1001/archinte.1993.00410180039004 8379800

[62] Hou L Huang Y Zhou R. Premenstrual syndrome is associated with altered cortisol awakening response. Stress. (2019) 22:640–6. 10.1080/10253890.2019.1608943 31057066

[63] Meng Y Huang D Hou L Zhou R. Hypoactivation of autonomic nervous system-related orbitofrontal and motor cortex during acute stress in women with premenstrual syndrome. Neurobiol Stress. (2021) 15:100357. 10.1016/j.ynstr.2021.100357 34258334 PMC8252112

[64] Yokoya S Maeno T Sakamoto N Goto R Maeno T. A brief survey of public knowledge and stigma towards depression. J Clin Med Res. (2018) 10:202. 10.14740/jocmr3282w 29416578 PMC5798266

[65] Arnaez JM Krendl AC McCormick BP Chen Z Chomistek AK. The association of depression stigma with barriers to seeking mental health care: A cross-sectional analysis. J Ment Health. (2020) 29:182–90. 10.1080/09638237.2019.1644494 31373519

[66] Pescosolido BA Halpern-Manners A Luo L Perry B. Trends in public stigma of mental illness in the US, 1996-2018. J Am Med Assoc Netw Open. (2021) 4:e2140202. 10.1001/jamanetworkopen.2021.40202 34932103 PMC8693212

[67] Ben-Zeev D Young MA. Accuracy of hospitalized depressed patients' and healthy controls' retrospective symptom reports: An experience sampling study. J Nerv Ment Dis. (2010) 198:280–5. 10.1097/NMD.0b013e3181d6141f 20386257

[68] Chan RCK Shi Y Lai M Wang Y Wang Y Kring AM. The Temporal Experience of Pleasure Scale (TEPS): Exploration and confirmation of factor structure in a healthy Chinese sample. PLoS ONE. (2012) 7:e35352. 10.1371/journal.pone.0035352 22530007 PMC3329425

[69] Hu MX Turner D Generaal E Bos D Ikram MK Ikram MA. Exercise interventions for the prevention of depression: A systematic review of meta-analyses. BMC Public Health. (2020) 20:1–11. 10.1186/s12889-020-09323-y 32811468 PMC7436997

[70] Liu Z Liu R Zhang Y Zhang R Liang L Wang Y. Association between perceived stress and depression among medical students during the outbreak of COVID-19: The mediating role of insomnia. J Affect Disord. (2021) 292:89–94. 10.1016/j.jad.2021.05.028 34107425 PMC8595067

[71] Sliwerski A Koszałkowska K. The influence of depression on biased diagnosis of premenstrual syndrome and premenstrual dysphoric disorder by the PSST inventory. Life. (2021) 11:1278. 10.3390/life11111278 34833154 PMC8624424

[72] Joyce KM Thompson K Good KP Tibbo PG O'Leary ME Perrot TS. The impact of depressed mood and coping motives on cannabis use quantity across the menstrual cycle in those with and without pre-menstrual dysphoric disorder. Addiction. (2021) 116:2746–58. 10.1111/add.15465 33651443

[73] Pearlstein T. Prevalence, impact on morbidity, and disease burden. In: PM O'Brien, A Rapkin, PJ Schmidt editors, The Premenstrual Syndromes: PMS and PMDD. 1st ed. London: CRC Press (2007). p. 10–1.

[74] Ismaili E Walsh S O'Brien PMS Bäckström T Brown C Dennerstein L. Fourth consensus of the International Society for Premenstrual Disorders (ISPMD): Auditable standards for diagnosis and management of premenstrual disorder. Arch Womens Ment Health. (2016) 19:953–8. 10.1007/s00737-016-0631-7 27378473

[75] Busse JW Montori VM Krasnik C Patelis-Siotis I Guyatt GH. Psychological intervention for premenstrual syndrome: A meta-analysis of randomized controlled trials. Psychother Psychosom. (2009) 78:6–15. 10.1159/000162296 18852497

[76] Soares CN Zitek B. Reproductive hormone sensitivity and risk for depression across the female life cycle: A continuum of vulnerability? J Psychiatry Neurosci. (2008) 33:331–43. 18592034 PMC2440795

[77] Panahi F Faramarzi M. The effects of mindfulness-based cognitive therapy on depression and anxiety in women with premenstrual syndrome. Depr Res Treat. (2016) 2016:9816481. 10.1155/2016/9816481 28025621 PMC5153465

