# Supplementary material for: The longitudinal predictive effect of self-reported frequency of premenstrual syndrome on depression: Findings from the Australian Longitudinal Study on Women's Health
Source: Front Public Health. 2023 Mar 23;11:1126190. doi: 10.3389/fpubh.2023.1126190 (PMC10076728; doi:10.3389/fpubh.2023.1126190)
Supplement: Supplementary file 1 [file Table_1.DOCX]

Supplementary Material

The longitudinal predictive effect of self-reported frequency of premenstrual syndrome on depression: Findings from the Australian Longitudinal Study on Women's Health

Lulu Hou, Lele Chen, Wenpei Zhang^*^

*** Correspondence:** Wenpei Zhang, zwpahut@ahut.edu.cn

**Table S1** Sample sizes of models in the logistic regression analysis

| Year | Dependent Variables | Model 1 | Model 2 | Model 3 | Model 4 | Model 5 |
| --- | --- | --- | --- | --- | --- | --- |
| **2003** | Diagnosis of depression in last 3 years | 6,490 | 5,749 | 5,530 | 5,530 | 5,510 |
|  | Depressive symptoms in last 12 months | 6,570 | 5,824 | 5,600 | 5,600 | 5,579 |
| **2006** | Diagnosis of depression in last 3 years | 6,118 | 5,477 | 5,266 | 5,266 | 5,245 |
|  | Depressive symptoms in last 12 months | 6,430 | 5,750 | 5,527 | 5,527 | 5,504 |
| **2009** | Diagnosis of depression in last 3 years | 5,352 | 4,850 | 4,675 | 4,675 | 4,657 |
|  | Depressive symptoms in last 12 months | 5,726 | 5,175 | 4,986 | 4,986 | 4,966 |
| **2012** | Diagnosis of depression in last 3 years | 5,586 | 5,058 | 4,877 | 4,877 | 4,860 |
|  | Depressive symptoms in last 12 months | 5,659 | 5,124 | 4,943 | 4,943 | 4,926 |
| **2015** | Diagnosis of depression in last 3 years | 5,104 | 4,628 | 4,464 | 4,464 | 4,446 |
|  | Depressive symptoms in last 12 months | 5,040 | 4,571 | 4,413 | 4,413 | 4,395 |
| **2018** | Diagnosis of depression in last 3 years | 4,908 | 4,428 | 4,270 | 4,270 | 4,256 |
|  | Depressive symptoms in last 12 months | 4,965 | 4,486 | 4,328 | 4,328 | 4,314 |

**Table S2** Cross-sectional prevalence (%) of self-reported diagnosis of depression, depressive symptoms, and PMS symptoms of the study sample at each survey

|  | 2000 (*n* = 9,688) | | 2003 (*n* = 9,081) | | 2006 (*n* = 9,145) | | 2009 (*n* = 8,199) | | 2012 (*n* = 8,009) | | 2015 (*n* = 7,186) | | 2018 (*n* = 7,121) | |
| --- | --- | --- | --- | --- | --- | --- | --- | --- | --- | --- | --- | --- | --- | --- |
|  | *n* | %^b^ | *n* | % ^b^ | *n* | % ^b^ | *n* | % ^b^ | *n* | % ^b^ | *n* | % ^b^ | *n* | % ^b^ |
| **Diagnosis of depression in last 3 years** ^a^ | | | | | | | | | | | | | | |
| Yes | 1,114 | 11.6 | 1,125 | 12.6 | 1,163 | 13.5 | 1339 | 17.7 | 1,340 | 17.1 | 1,123 | 15.9 | 1,068 | 15.6 |
| No | 8,471 | 88.4 | 7,810 | 87.4 | 7,449 | 86.5 | 6229 | 82.3 | 6,518 | 82.9 | 5,948 | 84.1 | 5,768 | 84.4 |
| No response | 103 |  | 146 |  | 533 |  | 631 |  | 151 |  | 115 |  | 285 |  |
| **Depressive symptoms in last 12 months** | | | | | | | | | | | | | | |
| Never | 6,679 | 69.1 | 5,819 | 64.3 | 5,837 | 64.5 | 5,155 | 63.8 | 4,600 | 57.8 | 3,752 | 53.9 | 3,554 | 51.4 |
| Rarely | 1,014 | 10.5 | 1,349 | 14.9 | 1,351 | 14.9 | 1,234 | 15.3 | 1,525 | 19.2 | 1,552 | 22.3 | 1,651 | 23.9 |
| Sometimes | 1,325 | 13.7 | 1,335 | 14.7 | 1,297 | 14.3 | 1,145 | 14.2 | 1,291 | 16.2 | 1,144 | 16.4 | 1,196 | 17.3 |
| Often | 653 | 6.8 | 553 | 6.1 | 564 | 6.2 | 544 | 6.7 | 540 | 6.8 | 518 | 7.4 | 509 | 7.4 |
| No response | 17 |  | 25 |  | 96 |  | 121 |  | 53 |  | 220 |  | 211 |  |
| **Premenstrual tension in last 12 months** | | | | | | | | | | | | | | |
| Never | 5,621 | 58.1 | 4,712 | 52.0 | 4,262 | 47.1 | 3,979 | 49.2 | 3,058 | 38.5 | 2,450 | 35.2 | 2,579 | 37.3 |
| Rarely | 848 | 8.8 | 1,327 | 14.7 | 1,620 | 17.9 | 1,408 | 17.4 | 1,684 | 21.2 | 1,579 | 22.7 | 1,582 | 22.9 |
| Sometimes | 1,931 | 20.0 | 2,001 | 22.1 | 2,128 | 23.5 | 1,865 | 23.1 | 2,214 | 27.8 | 2,030 | 29.1 | 1,963 | 28.4 |
| Often | 1,271 | 13.1 | 1,016 | 11.2 | 1,033 | 11.4 | 831 | 10.3 | 994 | 12.5 | 908 | 13.0 | 786 | 11.4 |
| No response | 17 |  | 25 |  | 102 |  | 116 |  | 59 |  | 219 |  | 211 |  |

^a^ In past 4 years for the 2000 survey.

^b^ Valid percentage of total responders at each survey.

**Table S3** The association between covariates and self-reported diagnosis of depression in Model 5

|  | 2003  (*n* = 5,510) | 2006  (*n* = 5,245) | 2009  (*n* = 4,657) | 2012  (*n* = 4,860) | 2015  (*n* = 4,446) | 2018  (*n* =4,256) |
| --- | --- | --- | --- | --- | --- | --- |
| **Age** (continuous) | 1.03 [0.96-1.11] | 0.94 [0.88-1.01] | 1.05 [0.99-1.12] | **1.07 [1.01-1.14]** | 1.03 [0.72-1.48] | 1.01 [0.94-1.07] |
| **Highest educational qualification** | | | | | | |
| Up to high school or equivalent | Reference | Reference | Reference | Reference | Reference | Reference |
| Trade/ diploma | 0.97 [0.74-1.27] | 0.92 [0.72-1.18] | 0.79 [0.63-1.00] | 0.97 [0.77-1.22] | 0.85 [0.67-1.10] | 0.90 [0.70-1.16] |
| University degree or higher | **0.70 [0.53-0.91]** | **0.62 [0.48-0.78]** | **0.66 [0.53-0.82]** | **0.81 [0.66-1.00]** | **0.75 [0.60-0.94]** | **0.72 [0.57-0.91]** |
| **Marital status** |  |  |  |  |  |  |
| Not married/separated/widowed | Reference | Reference | Reference | Reference | Reference | Reference |
| Married/de facto | 1.17 [0.93-1.46] | 0.92 [0.74-1.13] | **1.33 [1.10-1.60]** | 0.99 [0.83-1.19] | 1.00 [0.83-1.22] | 1.11 [0.90-1.35] |
| **Area of residence** |  |  |  |  |  |  |
| Major cities | Reference | Reference | Reference | Reference | Reference | Reference |
| Inner regional | 1.02 [0.80-1.30] | 0.98 [0.78-1.23] | **1.24 [1.01-1.51]** | 1.07 [0.88-1.30] | 1.08 [0.88-1.34] | 1.14 [0.92-1.42] |
| Outer regional/remote/very remote | 0.86 [0.63-1.18] | 1.11 [0.85-1.46] | 0.95 [0.74-1.64] | 1.03 [0.80-1.31] | 1.04 [0.80-1.35] | 0.93 [0.70-1.22] |
| **BMI** |  |  |  |  | ^*^ |  |
| Underweight [<18.5] | 0.98 [0.62-1.55] | 0.85 [0.54-1.33] | 1.00 [0.68-1.47] | 0.95 [0.65-1.39] | 0.83 [0.54-1.26] | 0.93 [0.61-1.42] |
| Normal weight [18.5 to <25] | Reference | Reference | Reference | Reference | Reference | Reference |
| Overweight [25 to <30] | 1.00 [0.76-1.32] | 1.04 [0.81-1.34] | 0.90 [0.71-1.14] | 1.04 [0.84-1.30] | 1.23 [0.98-1.55] | 1.16 [0.91-1.48] |
| Obese [≥30] | 1.09 [0.78-1.51] | **1.55 [1.16-2.06]** | 1.24 [0.94-1.64] | **1.64 [1.17-2.12]** | **1.69 [1.29-2.22]** | **1.89 [1.44-2.49]** |
| Sleeping Difficulties in the last 12 months |  |  |  |  |  |  |
| Never | Reference | Reference | Reference | Reference | Reference | Reference |
| Rarely | **1.54 [1.08-2.21]** | 1.19 [0.85-1.68] | 1.00 [0.73-1.36] | 1.13 [0.85-1.50] | 0.76 [0.55-1.05] | 0.93 [0.67-1.29] |
| Sometimes | **1.96 [1.49-2.57]** | **1.78 [1.38-2.29]** | **1.43 [1.14-1.81]** | 1.22 [0.97-1.53] | 1.17 [0.91-1.49] | 1.13 [0.87-1.47] |
| Often | **2.00 [1.42-2.81]** | **2.18 [1.59-2.97]** | **1.42 [1.04-1.94]** | 1.16 [0.85-1.57] | 0.87 [0.62-1.23] | 1.23 [0.88-1.73] |
| **Physical activity** |  |  |  |  |  |  |
| Sedentary | Reference | Reference | Reference | Reference | Reference | Reference |
| Low | 0.83 [0.56-1.23] | 1.04 [0.72-1.50] | 1.07 [0.76-1.50] | 0.77 [0.57-1.05] | 0.89 [0.64-1.24] | 1.00 [0.71-1.41] |
| Moderate | 0.79 [0.53-1.20] | 0.94 [0.64-1.38] | 0.94 [0.66-1.34] | **0.70 [0.51-0.97]** | **0.66 [0.46-0.94]** | 0.70 [0.49-1.01] |
| High | 0.98 [0.67-1.45] | 1.06 [0.74-1.53] | 0.86 [0.61-1.22] | **0.67 [0.49-0.91]** | **0.69 [0.49-0.97]** | 0.79 [0.56-1.12] |
| **Smoking** |  |  |  |  |  |  |
| Never smoker | Reference | Reference | Reference | Reference | Reference | Reference |
| Ex-smoker | 1.05 [0.77-1.45] | 1.25 [0.94-1.67] | 1.24 [0.96-1.61] | 1.08 [0.83-1.39] | **1.54 [1.20-1.98]** | 1.14 [0.87-1.51] |
| Current smoker | 1.25 [0.98-1.61] | **1.34 [1.06-1.69]** | **1.29 [1.05-1.60]** | 1.21 [0.99-1.49] | 1.10 [0.87-1.37] | **1.26 [1.05-1.53]** |
| **Alcohol consumption** |  |  |  |  |  |  |
| Never drinker/rarely drinks | Reference | Reference | Reference | Reference | Reference | Reference |
| Low risk drinker | 0.89 [0.71-1.12] | **0.74 [0.60-0.91]** | 1.01 [0.83-1.24] | 0.83 [0.69-1.00] | 0.83 [0.68-1.01] | 0.86 [0.70-1.06] |
| Risky/high risk drinker | 0.81 [0.45-1.66] | 0.88 [0.53-1.47] | **1.62 [1.06-2.49]** | 0.65 [0.40-1.05] | 0.84 [0.51-1.39] | **0.53 [0.30-0.95]** |
| **OCPs use** |  |  |  |  |  |  |
| Not using OCPs | Reference | Reference | Reference | Reference | Reference | Reference |
| Using OCPs | 1.03 [0.82-1.28] | 0.95 [0.77-1.16] | 0.91 [0.76-1.10] | 1.00 [0.84-1.20] | 0.72 [0.80-1.17] | 1.02 [0.83-1.24] |
| **Irregular periods** |  |  |  |  |  |  |
| Never | Reference | Reference | Reference | Reference | Reference | Reference |
| Rarely | 1.06 [0.69-1.63] | 0.66 [0.43-1.01] | 0.80 [0.54-1.19] | 1.20 [0.85-1.69] | 1.04 [0.470-1.54] | 0.98 [0.65-1.47] |
| Sometimes | **1.41 [1.01-1.97]** | 1.33 [0.97-1.81] | 1.30 [0.98-1.74] | 1.16 [0.87-1.54] | 1.09 [0.80-1.49] | 1.22 [0.88-1.68] |
| Often | 1.06 [0.72-1.56] | 1.06 [0.75-1.50] | 1.05 [0.76-1.45] | 1.11 [0.81-1.51] | 1.31 [0.94-1.82] | 1.07 [0.75-1.52] |
| **Heavy periods** |  |  |  |  |  |  |
| Never | Reference | Reference | Reference | Reference | Reference | Reference |
| Rarely | 0.73 [0.44-1.18] | 1.16 [0.75-1.78] | 1.05 [0.69-1.59] | 0.81 [0.54-1.22] | 1.00 [0.65-1.56] | 0.66 [0.41-1.06] |
| Sometimes | 0.86 [0.58-1.26] | 0.77 [0.54-1.12] | **0.65 [0.48-0.93]** | 0.85 [0.61-1.17] | 0.96 [0.68-1.35] | **0.57 [0.38-0.84]** |
| Often | 0.75 [0.47-1.22] | 0.84 [0.55-1.30] | 0.92 [0.60-1.40] | 1.38 [0.96-1.98] | 1.00 [0.66-1.52] | 0.93 [0.60-1.44] |
| **Severe period pain** |  |  |  |  |  |  |
| Never | Reference | Reference | Reference | Reference | Reference | Reference |
| Rarely | 1.02 [0.68-1.54] | 1.09 [0.75-1.57] | 1.16 [0.83-1.61] | 1.15 [0.85-1.57] | 1.37 [0.99-1.90] | 1.04 [0.73-1.48] |
| Sometimes | 1.03 [0.74-1.44] | 1.12 [0.83-1.53] | 1.08 [0.81-1.43] | 1.12 [0.86-1.46] | 1.00 [0.74-1.35] | 1.01 [0.75-1.38] |
| Often | 1.40 [0.952.05] | 1.14 [0.79-1.63] | 1.11 [0.79-1.57] | 0.88 [0.63-1.23] | 0.89 [0.62-1.28] | 0.75 [0.51-1.11] |
| History of abuse |  |  |  |  |  |  |
| No | Reference | Reference | Reference | Reference | Reference | Reference |
| Yes | **1.66 [1.33-2.08]** | **1.36 [1.11-1.67]** | **1.60 [1.33-1.93]** | **1.47 [1.23-1.76]** | **1.54 [1.27-1.87]** | **1.42 [1.16-1.73]** |
| **Perceived stress** |  |  |  |  |  |  |
| Not at all/somewhat stressed | Reference | Reference | Reference | Reference | Reference | Reference |
| Moderately stressed | **1.78 [1.41-2.25]** | **1.61[1.30-1.99]** | **1.55 [1.28-1.88]** | **1.52 [1.27-1.83]** | **1.68 [1.44-3.12]** | **1.51 [1.23-1.85]** |
| Very/extremely stressed | **3.14 [2.13-4.64]** | **2.30 [1.58-3.35]** | **1.74 [1.18-2.57]** | **2.11 [1.48-3.01]** | **2.12 [1.44-3.12]** | **2.09 [1.39-3.14]** |

Note. Significant ORs are in bold.

**Table S4** The association between covariates and self-reported frequency of depressive symptoms in Model 5

|  | 2003  (*n* = 5,579) | 2006  (*n* = 5,504) | 2009  (*n* = 4,966) | 2012  (*n* = 4,926) | 2015  (*n* = 4,395) | 2018  (*n* =4,314) |
| --- | --- | --- | --- | --- | --- | --- |
| **Age** (continuous) | 1.02 [0.98-1.06] | **1.06 [1.02-1.10]** | **1.05 [1.00-1.09]** | **1.06 [1.02-1.11]** | 1.00 [0.96-1.05] | 1.04 [1.00-1.08] |
| **Highest educational qualification** | | | | | | |
| Up to high school or equivalent | Reference | Reference | Reference | Reference | Reference | Reference |
| Trade/ diploma | 1.01 [0.86-1.17] | 1.06 [0.91-1.23] | **0.85 [0.72-1.00]** | 0.90 [0.77-1.06] | 0.88 [0.75-1.04] | **0.82 [0.69-0.96]** |
| University degree or higher | **0.75 [0.65-0.87]** | **0.72 [0.62-0.83]** | **0.78 [0.67-0.90]** | **0.83 [0.72-0.96]** | **0.70 [0.61-0.81]** | **0.69 [0.59-0.79]** |
| **Marital status** |  |  |  |  |  |  |
| Not married/separated/widowed | Reference | Reference | Reference | Reference | Reference | Reference |
| Married/de facto | 0.93 [0.83-1.06] | 1.03 [0.91-1.17] | **1.28 [1.13-1.46]** | **1.20 [1.06-1.35]** | **1.18 [1.04-1.34]** | **1.16 [1.01-1.33]** |
| **Area of residence** |  |  |  |  |  |  |
| Major cities | Reference | Reference | Reference | Reference | Reference | Reference |
| Inner regional | 1.06 [0.92-1.21] | 1.10 [0.96-1.25] | 1.12 [0.98-1.29] | 1.12 [0.98-1.27] | 0.99 [0.86-1.14] | **1.16 [1.01-1.33]** |
| Outer regional/remote/very remote | 1.06 [0.90-1.25] | 1.15 [0.97-1.35] | **1.19 [1.01-1.41]** | 1.04 [0.88-1.22] | 0.88 [0.74-1.04] | 1.12 [0.94-1.32] |
| **BMI** |  |  |  |  |  |  |
| Underweight [<18.5] | 0.95 [0.74-1.20] | 1.05 [0.82-1.34] | 1.16 [0.94-1.42] | 1.17 [0.91-1.49] | 1.14 [0.89-1.47] | 1.04 [0.81-1.33] |
| Normal weight [18.5 to <25] | Reference | Reference | Reference | Reference | Reference | Reference |
| Overweight [25 to <30] | **0.80 [0.69-0.93]** | 1.00 [0.86-1.16] | 0.95 [0.81-1.11] | 1.08 [0.93-1.26] | **1.21 [1.04-1.41]** | 1.04 [0.90-1.21] |
| Obese [≥30] | 0.85 [0.70-1.04] | 1.03 [0.84-1.25] | 1.16 [0.94-1.41] | **1.42 [1.17-1.72]** | **1.56 [1.28-1.90]** | **1.38 [1.13-1.69]** |
| Sleeping Difficulties in the last 12 months |  |  |  |  |  |  |
| Never | Reference | Reference | Reference | Reference | Reference | Reference |
| Rarely | **1.27 [1.04-1.54]** | **1.36 [1.12-1.65]** | **1.27 [1.04-1.56]** | 1.13 [0.93-1.37] | 1.08 [0.88-1.32] | 1.14 [0.93-1.39] |
| Sometimes | **1.68 [1.44-1.96]** | **1.73 [1.48-2.02]** | **1.59 [1.35-1.87]** | **1.43 [1.22-1.68]** | **1.41 [1.19-1.65]** | **1.39 [1.18-1.64]** |
| Often | **1.85 [1.51-2.28]** | **1.93 [1.57-2.38]** | **1.34 [1.07-1.69]** | **1.46 [1.17-1.82]** | **1.43 [1.14-1.80]** | **1.77 [1.41-2.23]** |
| **Physical activity** |  |  |  |  |  |  |
| Sedentary | Reference | Reference | Reference | Reference | Reference | Reference |
| Low | 0.94 [0.75-1.17] | 1.10 [0.88-1.38] | 0.82 [0.65-1.04] | 0.94 [0.75-1.18] | 0.98 [0.77-1.24] | 0.87[0.69-1.09] |
| Moderate | 0.91 [0.72-1.15] | 0.96 [0.76-1.22] | **0.75 [0.59-0.95]** | 0.83 [0.65-1.05] | **0.69 [0.54-0.88]** | **0.69 [0.55-0.88]** |
| High | 0.88 [0.70-1.10] | 0.94 [0.75-1.18] | **0.68 [0.54-0.86]** | 0.85 [0.67-1.06] | **0.78 [0.61-0.98]** | **0.73 [0.58-0.91]** |
| **Smoking** |  |  |  |  |  |  |
| Never smoker | Reference | Reference | Reference | Reference | Reference | Reference |
| Ex-smoker | 1.15 [0.97-1.37] | 1.03 [0.86-1.23] | 1.16 [0.96-1.39] | **1.26 [1.06-1.50]** | 1.19 [0.99-1.42] | 1.14 [0.95-1.36] |
| Current smoker | **1.31 [1.14-1.50]** | **1.20 [1.04-1.39]** | **1.19 [1.03-1.38]** | **1.17 [1.01-1.35]** | 1.04 [0.90-1.21] | **1.36 [1.18-1.58]** |
| **Alcohol consumption** |  |  |  |  |  |  |
| Never drinker/rarely drinks | Reference | Reference | Reference | Reference | Reference | Reference |
| Low risk drinker | 1.01 [0.89-1.15] | 1.00 [0.88-1.14] | 0.95 [0.83-1.09] | **0.82 [0.72-0.93]** | **0.87 [0.76-1.00]** | 0.89 [0.78-1.02] |
| Risky/high risk drinker | 1.05 [0.76-1.45] | **1.41 [1.03-1.92]** | 1.28 [0.93-1.77] | 0.96 [0.70-1.31] | 1.02 [0.74-1.42] | 0.88 [0.63-1.23] |
| **OCPs use** |  |  |  |  |  |  |
| Not using OCPs | Reference | Reference | Reference | Reference | Reference | Reference |
| Using OCPs | **0.87 [0.77-0.99]** | **0.87 [0.77-0.99]** | **0.86 [0.76-0.98]** | 0.92 [0.81-1.04] | 0.94 [0.83-1.07] | 0.89 [0.78-1.01] |
| **Irregular periods** |  |  |  |  |  |  |
| Never | Reference | Reference | Reference | Reference | Reference | Reference |
| Rarely | 1.04 [0.82-1.32] | 0.96 [0.76-1.22] | 1.11 [0.87-1.43] | 1.15 [0.90-1.46] | 0.95 [0.74-1.24] | 0.80 [0.62-1.04] |
| Sometimes | **1.33 [1.10-1.62]** | 1.22 [1.00-1.49] | **1.27 [1.03-1.57]** | 1.13 [0.92-1.39] | 1.08 [0.88-1.33] | 0.96 [0.78-1.19] |
| Often | 1.14 [0.92-1.42] | **1.25 [1.01-1.55]** | 1.26 [1.00-1.58] | 1.21 [0.97-1.51] | 1.21 [0.97-1.52] | 1.25 [0.99-1.56] |
| **Severe period pain** |  |  |  |  |  |  |
| Never | Reference | Reference | Reference | Reference | Reference | Reference |
| Rarely | 0.93 [0.71-1.22] | 0.93 [0.71-1.23] | 0.94 [0.71-1.26] | **0.74 [0.56-0.98]** | 0.93 [0.69-1.25] | 0.97 [0.73-1.29] |
| Sometimes | 0.93 [0.74-1.16] | 0.97 [0.77-1.21] | **0.72 [0.56-0.91]** | 0.90 [0.72-1.13] | 1.01 [0.80-1.28] | 0.99 [0.78-1.25] |
| Often | 1.15 [0.88-1.51] | 0.99 [0.76-1.31] | 1.08 [0.81-1.45] | 1.06 [0.80-1.40] | **1.41 [1.06-1.87]** | 0.97 [0.72-1.31] |
| **Heavy periods** |  |  |  |  |  |  |
| Never | Reference | Reference | Reference | Reference | Reference | Reference |
| Rarely | 1.02 [0.82-1.26] | 1.10 [0.88-1.37] | 1.19 [0.96-1.48] | **1.34 [1.09-1.65]** | 1.08 [0.87-1.35] | 1.03 [0.83-1.29] |
| Sometimes | 0.92 [0.76-1.11] | 1.14 [0.94-1.38] | 0.93 [0.76-1.13] | 1.04 [0.86-1.26] | 1.02 [0.84-1.23] | 1.12 [0.93-1.37] |
| Often | 0.86 [0.68-1.09] | 0.98 [0.78-1.24] | 0.93 [0.73-1.19] | 1.02 [0.81-1.29] | 0.80 [0.63-1.02] | 0.98 [0.77-1.24] |
| History of abuse |  |  |  |  |  |  |
| No | Reference | Reference | Reference | Reference | Reference | Reference |
| Yes | **1.51 [1.33-1.70]** | **1.35 [1.19-1.53]** | **1.51 [1.33-1.72]** | **1.33 [1.18-1.50]** | **1.35 [1.19-1.54]** | **1.24 [1.09-1.41]** |
| **Perceived stress** |  |  |  |  |  |  |
| Not at all/somewhat stressed | Reference | Reference | Reference | Reference | Reference | Reference |
| Moderately stressed | **1.79 [1.58-2.03]** | **1.86 [1.64-2.11]** | **1.74 [1.53-1.99]** | **1.71 [1.51-1.94]** | **1.71 [1.51-1.95]** | **1.67 [1.46-1.90]** |
| Very/extremely stressed | **3.22 [2.48-4.19]** | **2.56 [1.97-3.33]** | **2.23 [1.68-2.95]** | **2.75 [2.09-3.61]** | **2.40 [1.80-3.21]** | **2.56 [1.92-3.42]** |

Note. Significant ORs are in bold.

**Table S5** The adjusted models for self-reported diagnosis of depression using multiple imputation for missing data of covariates (*p*-values)

| PMS in 2000 | 2003  (*n* = 6,490) | 2006  (*n* = 6,118) | 2009  (*n* = 5,352) | 2012  (*n* = 5,586) | 2015  (*n* = 5,104) | 2018  (*n* =4,908) |
| --- | --- | --- | --- | --- | --- | --- |
| Never | Reference | Reference | Reference | Reference | Reference | Reference |
| Rarely | .57 | .68 | .10 | .40 | .53 | .08 |
| Sometimes | .47 | **.05** | .22 | **.02** | .15 | **.003** |
| Often | .12 | **.005** | .23 | **< .001** | **.02** | **.001** |

**Table S6** The adjusted models for self-reported frequency of depressive symptoms using multiple imputation for missing data of covariates (*p*-values)

| PMS in 2000 | 2003  (*n* = 6,570) | 2006  (*n* = 6,430) | 2009  (*n* = 5,726) | 2012  (*n* = 5,659) | 2015  (*n* = 5,040) | 2018  (*n* =4,965) |
| --- | --- | --- | --- | --- | --- | --- |
| Never | Reference | Reference | Reference | Reference | Reference | Reference |
| Rarely | .47 | .83 | .53 | .99 | .48 | .89 |
| Sometimes | **< .001** | **.003** | **.002** | **< .001** | **.001** | **.01** |
| Often | **< .001** | **< .001** | **< .001** | **< .001** | **< .001** | **< .001** |
